# Complete plastome sequence of *Magnolia omeiensis* (W.C. Cheng) Dandy (Magnoliaceae)

**DOI:** 10.1080/23802359.2019.1660596

**Published:** 2019-09-02

**Authors:** Guo-Qing Wang, Xiao-Feng Zhang, Hong-Xin Wang, Zhi-Xin Zhu, Hua-Feng Wang

**Affiliations:** Hainan Key Laboratory for Sustainable Utilization of Tropical Bioresources, School of Life and Pharmaceutical Sciences, Hainan University, Haikou, China

**Keywords:** *Magnolia omeiensis*, plastome, phylogeny, genome structure, Magnoliaceae

## Abstract

*Magnolia omeiensis* is an evergreen tree with 25 meters heights. It is distributed in tropical 1200–1300 m. Mount Emei. And it has been ranked as a CR (Critically Endangered) species in China. Here, we report and characterize the complete plastome of *M. omeiensis* in an effort to provide genomic resources useful for promoting its systematics research. The plastome of *M. omeiensis i*s found to possess a total length 160,021 bp with the typical quadripartite structure of angiosperms, contains two Inverted Repeats (IRs) of 26,336 bp, a Large Single-Copy (LSC) region of 88,061 bp and a Small Single-Copy (SSC) region of 19,288 bp. The plastome contains 114 genes, consisting of 80 unique protein-coding genes, 30 unique tRNA genes and 4 unique rRNA genes. The overall A/T content in the plastome of *M. omeiensis* is 60.70%. The phylogenetic analysis indicated that *M. omeiensis* is close to *Magnolia yunnanensis* within Magnoliaceae in this study. The complete plastome sequence of *M. omeiensis* will provide a useful resource for the conservation genetics of this species as well as for the phylogenetic studies of Magnoliaceae.

## Introduction

*Magnolia omeiensis* (W.C.Cheng) Dandy (Magnoliaceae, Magnolia) grows as an evergreen tree that branches and leaves are luxuriant. It easily reaches heights of 25 meters and trunk diameters of 40 centimeters. It is distributed in 1200–1300 m. Mount Emei (Nianhe et al. 2008). It has been ranked as a CR (Critically Endangered) species in China (Qin et al. 2017). Consequently, the genetic and genomic information is urgently needed to promote its systematics research and the development of conservation value of *M. omeiensis.* Here, we report and characterize the complete plastome of *M. omeiensis* (GenBank accession number: MN197534). This is the first report of a complete plastome for *M. omeiensis.*

In this study, *M. omeiensis* was sampled from the Mount Emei in Sichuan Province of China (N29.560°, E103.355°). A voucher specimen (Wang et al., B150) was deposited in the Herbarium of the Institute of Tropical Agriculture and Forestry (HUTB), Hainan University, Haikou, China.

The experiment procedure is as reported in Zhu et al. (2018). Around 6 Gb clean data were assembled against the plastome of *Cryptocarya chinensis* (NC_036002.1) (Wu et al. 2017) using MITObim v1.8 (Hahn et al. 2013). The plastome was annotated using Geneious R8.0.2 (Biomatters Ltd., Auckland, New Zealand) against the plastome of *C. chinensis* (NC_036002.1). The annotation was corrected with DOGMA (Wyman et al. 2004).

The plastome of *M. omeiensis* is found to possess a total length 160,021 bp with the typical quadripartite structure of angiosperms, contains two Inverted Repeats (IRs) of 26,336 bp, a Large Single-Copy (LSC) region of 88,061 bp and a Small Single-Copy (SSC) region of 19,288 bp. The plastome contains 114 genes, consisting of 80 unique protein-coding genes, 30 unique tRNA genes and 4 unique rRNA genes. The overall A/T content in the plastome of *M. omeiensis* is 60.70%, which the corresponding value of the LSC, SSC and IR region were 62.00%, 65.80% and 56.80%, respectively.

**Figure 1.**
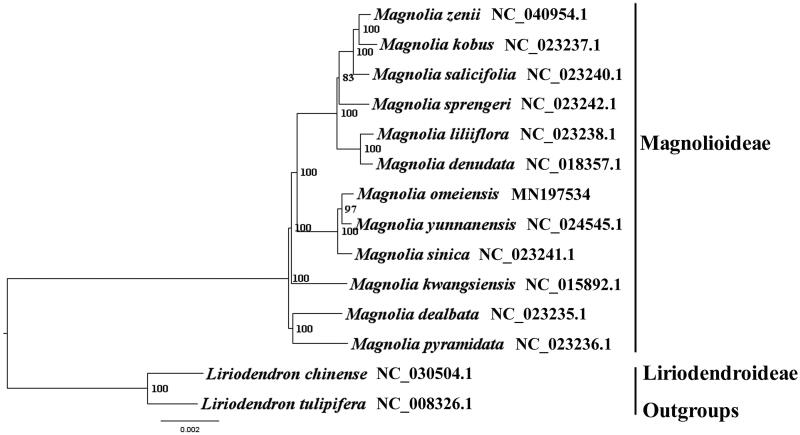
The best ML phylogeny recovered from 14 complete plastome sequences by RAxML. Accession numbers: *Magnolia omeiensis* MN197534, *Magnolia pyramidata* NC_023236.1, *Magnolia dealbata* NC_023235.1, *Magnolia kwangsiensis* NC_015892.1, *Magnolia sinica* NC_023241.1, *Magnolia yunnanensis* NC_024545.1, *Magnolia zenii* NC_040954.1*, Magnolia Kobus* NC_023237.1, *Magnolia salicifolia* NC_023240.1, *Magnolia sprengeri* NC_023242.1, *Magnolia liliiflora* NC_023238.1, *Magnolia denudate* NC_018357.1, Outgroups: *Liriodendron chinense* NC_030504.1, *Liriodendron tulipifera* NC_008326.1.

We used RAxML (Stamatakis 2006) with 1000 bootstraps under the GTRGAMMAI substitution model to reconstruct a maximum likelihood (ML) phylogeny of 11 published complete plastomes of Magnolioideae, using 2 species of Liriodendroideae as outgroups. The phylogenetic analysis indicated that *M. omeiensis* is close to *Magnolia yunnanensis* within Magnoliaceae in this study (Figure 1). Most nodes in the plastome ML tree were strongly supported. The complete plastome sequence of *M. omeiensis* will provide a useful resource for the conservation genetics of this species as well as for the phylogenetic studies of Magnoliaceae.
